# Maternal control during prenatal oxycodone exposure: differential effects of voluntary versus involuntary self-administration on offspring substance use vulnerability in rats

**DOI:** 10.1038/s41386-026-02422-1

**Published:** 2026-05-04

**Authors:** Chantal CA Aaron, Kerri E. Budge, Sara B. Isgate, Fair M. Vassoler, Elizabeth M. Byrnes

**Affiliations:** Department of Comparative Pathobiology, Cummings School of Veterinary Medicine, Tufts University, Grafton, MA USA

**Keywords:** Addiction, Developmental biology

## Abstract

Prenatal opioid exposure is a growing clinical concern, yet the impact of maternal control over drug intake on long-term offspring outcomes remains unclear. Here, we utilized a translational rat model to examine how the mode of oxycodone administration during pregnancy influences substance use vulnerability in adult offspring. Female Sprague-Dawley rats (*n* = 10-11/group) were assigned to one of three groups: voluntary intravenous self-administration (Oxycodone-Lead), yoked non-contingent oxycodone delivery (Oxycodone-Yoked), or yoked saline control (Saline-Yoked). The dam self-administered oxycodone in 6h sessions for three weeks, 5 days/week, prior to conception and then daily throughout gestation. Offspring were cross-fostered to drug-naïve dams at birth. In adulthood, male and female offspring were evaluated for oxycodone (0.1 mg/kg/infusion) and cocaine (0.5 mg/kg/infusion) intravenous self-administration using fixed ratio (7 sessions FR1; 5 sessions FR5) and progressive ratio schedules (3 sessions), as well as drug-seeking under extinction conditions (1 session). Despite equivalent overall drug exposure, offspring of Oxycodone-Yoked dams exhibited enhanced motivated responding and drug-seeking behaviors. Female Oxycodone-Yoked offspring showed increased intake and motivated responding specifically for oxycodone, while Oxycodone-Yoked offspring of both sexes displayed augmented cocaine seeking and drug-seeking under extinction. Pearson’s correlations between maternal intake and offspring behavior were sex- and drug-specific and were only observed in the Oxycodone-Yoked group. These findings indicate that maternal control over drug intake, rather than opioid exposure per se, plays a critical role in shaping neurodevelopmental trajectories underlying substance use vulnerability. This study highlights the importance of considering patterns of maternal opioid use when assessing long-term developmental risks.

## Introduction

Based on 2019 surveillance data from the United States, 7% of pregnant women use prescription opioids, with 1 in 5 reporting misuse [1]. Although prescribing of the mu-opioid receptor agonist oxycodone has declined substantially from its peak around 2010 to 2012 [2], it still remains one of the most prescribed opioids in the US [3]. Moreover, individuals prenatally exposed during peak prescribing years are now reaching adolescence and adulthood. While clinical data on adult outcomes following prenatal oxycodone exposure are limited, preclinical models can identify areas of concern as well as potential mechanisms underlying effects in offspring. Rodent studies indicate that prenatal opioids impact reward-related brain development and increase substance use vulnerability in offspring [4–6]. Most findings use morphine as a prototypical opioid, and despite variability in timing, route, and dose, the confluence of findings strongly suggests that perinatal opioids impact substance use vulnerability [4–6]. Whether these effects are due to direct fetal exposure, alterations in maternal physiology during gestation, and/or maternal care postpartum remains unclear.

Recently, the number of preclinical studies examining the impact of prenatal exposure to oxycodone has increased [7]. While some studies have examined long-term outcomes [8, 9], there are limited data regarding potential substance use vulnerability in oxycodone-exposed offspring [10]. To address this gap, we implemented a model of prenatal oxycodone exposure to examine substance use vulnerability in adult offspring. All subjects were reared by drug-naïve dams to limit the impact of opioid-induced changes in maternal care [11].

Most prenatal oxycodone studies utilize non-contingent, experimenter administration [8, 10, 12, 13]. There is evidence, however, that the mode of drug administration, whether voluntary or involuntary, can produce distinct neurobiological and behavioral effects. For example, lack of control over drug intake is associated with increased stress responses, altered dopaminergic and glutamatergic signaling, and aversive responses to drugs [14–17]. Moreover, non-contingent administration has been shown to differentially impact offspring in a male preconception model [18]. Given these findings, outcomes observed in experimenter-administered studies may not be directly comparable to those in which the subject has control over their intake.

To address this question, we used a long access (6 h/day) intravenous self-administration model (IVSA) of prenatal oxycodone exposure. The benefits of the IV route of administration are twofold; first, it avoids significant species differences in oxycodone metabolism and bioavailability [19], and second, it allows for the investigation of contingent versus non-contingent drug delivery. Thus, in the current model, every self-administering oxycodone female was yoked to a female receiving a non-contingent saline infusion (Saline-Yoked) and another female receiving a non-contingent oxycodone infusion (Oxycodone-Yoked). Our goal was to determine whether contingent or non-contingent oxycodone intake during pregnancy influenced substance use vulnerability in adulthood. Thus, as adults, male and female offspring were tested using IVSA for either oxycodone or cocaine. Using 2 h sessions, we examined acquisition under a fixed ratio 1 schedule (FR1), effort/motivation under both FR5 and progressive ratio (PR), and initial drug seeking on day 1 of extinction. Responses were then correlated with average maternal intake during pregnancy.

## Methods

### Subjects

Nulliparous female Sprague-Dawley rats (225–250 g) were purchased from Charles River Breeding Laboratories (Kingston, NU, USA) and housed in a light- (0700–1900 h), temperature- (21–24 °C), and humidity- (45–55%) controlled environment in standard polycarbonate cages. Food and water were available *ad libitum*. Experiments were performed in accordance with the Guide for the Care and Use of Laboratory Animals (NIH) with approval from the Institutional Animal Care and Use Committee at Tufts University.

### Intrajugular catheterization

One week after arrival, females were anesthetized with a ketamine/dexmedetomidine (66.67 mg/kg and 0.167 mg/kg, respectively) and surgically implanted with intrajugular catheters (SAI Infusion Technologies, Lake Villa, IL) fed into the right external jugular vein and routed to a mesh back mount platform secured subcutaneously between the scapulae. Catheters were flushed daily with 0.3 ml of Baytril (2.27 mg/mL) in heparinized saline (20 IU/mL in 0.9% saline). All females were individually housed following surgery and throughout self-administration.

### Preconception and gestational self-administration

**Fig. 1:**
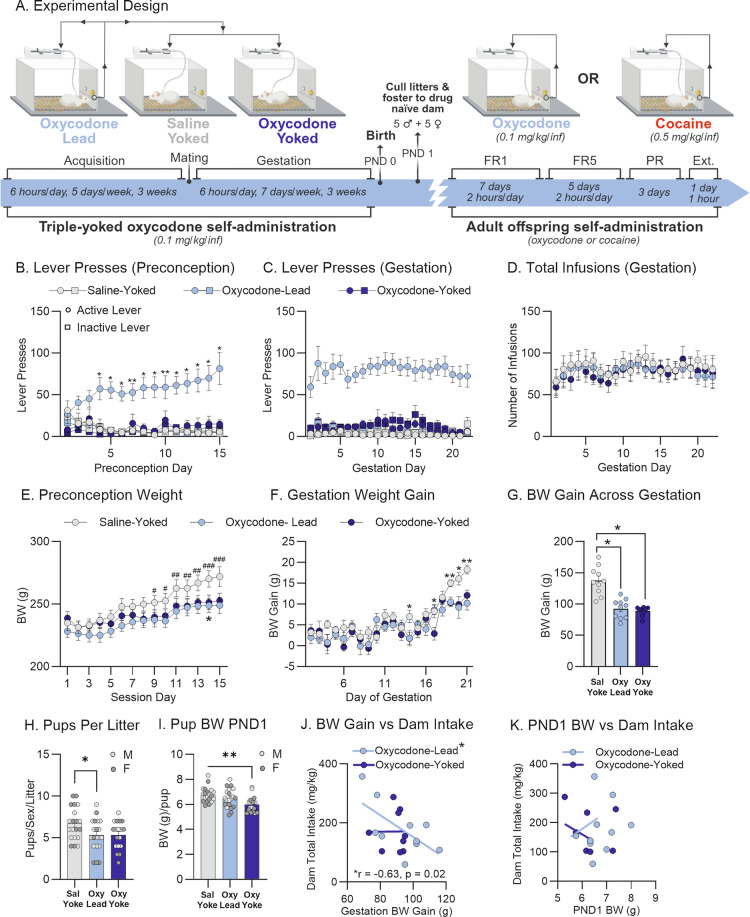
Experimental design, gestational self-administration, and postnatal outcomes. **A** Schematic of the experimental design. Female rats were assigned to Oxycodone-Lead, Oxycodone-Yoked, or Saline-Yoked groups and underwent intravenous self-administration (IVSA) for 3 weeks prior to mating and throughout gestation. Offspring were cross-fostered to drug-naïve dams at PND1 and tested as adults for oxycodone or cocaine IVSA. **B** Active and inactive lever presses during preconception acquisition. Oxycodone-Lead females demonstrated significant active versus inactive lever discrimination beginning on session 4. **C** Active and inactive lever presses across gestation. Oxycodone-Lead females maintained significantly higher active lever pressing compared to yoked groups throughout pregnancy. **D** Total infusions received during gestation did not differ between groups. **E** Body weight across preconception sessions. Both oxycodone groups weighed significantly less than Saline-Yoked females by mating. **F** Daily gestational weight gain and **G** total gestational weight gain were significantly reduced in both oxycodone groups compared to Saline-Yoked. **H** Litter size and **I** individual pup body weight on PND1. Oxycodone-Yoked pups were significantly smaller than Saline-Yoked pups. **J** Correlation between dam total gestational oxycodone intake and gestational body weight gain in Oxycodone-Lead and Oxycodone-Yoked dams. **K** Correlation between dam total oxycodone intake and offspring body weight on PND1. Data are expressed as mean ± SEM. *p < 0.05, **p < 0.01, ###*p* < 0.001 vs. Saline-Yoked.

The full experimental design is shown in Fig. 1A. One week after surgery, IVSA training was initiated in operant chambers housed within sound-attenuating cubicles (Med Associates, St. Albans, VT) equipped with house and cue lights, as well as two retractable levers (one active and one inactive). Upon an active lever press, subjects received a 60 μl infusion (0.1 mg/kg oxycodone or saline) over 3–5 s (based on syringe size) with 15 s post-infusion timeout. Sessions began between 0800 and 1000 during the light cycle. We used a triple-yoke design where the operant chamber of each female self-administering oxycodone (Oxycodone-Lead) was linked to two other chambers. In one of the chambers, the female would receive an infusion of saline matched to the Oxycodone-Lead, while in the other chamber, the female would receive an infusion of oxycodone to match the leader. Preconception self-administration consisted of 15 days of 6 h sessions on FR1 (5 days/week). Females were then mated (see details below), after which they were run daily (6 h/session) for 21 days. All females were weighed daily to adjust drug concentration based on weight.

### Breeding, postpartum assessment and cross-fostering

After the initial 15 days of self-administration, females were injected with gonadotropin-releasing hormone (40 µg in 0.2 ml; Sigma-Aldrich, St. Louis) to ensure timed mating with drug-naïve males. Pregnancy was determined by the presence of sperm using vaginal lavage, which was designated as gestation day 0 (GD0). Self-administration continued daily throughout pregnancy until gestation day 21 (the day before parturition). Following parturition, on PND1, litters were weighed and culled to a maximum of five males/five females. Each litter was then assigned to a drug naïve lactating donor dam, as our previous studies revealed effects on maternal retrieval in oxycodone self-administering dams [20]. On PND21, offspring were weaned from their dams and group-housed with same sex littermates until adult catheter implantation, after which they were individually housed.

A total of 50 females underwent surgery and initiated self-administration training. Of these, 32 met criteria for inclusion, defined as maintaining catheter patency throughout the study, confirmed pregnancy via vaginal lavage, and delivery of a litter with a minimum of 6 pups. Attrition was higher in oxycodone-exposed groups (Oxycodone-Lead: *n* = 8 excluded; Oxycodone-Yoked: *n* = 9 excluded) compared to Saline-Yoked controls (*n* = 1 excluded), primarily due to catheter failure and pregnancy loss, consistent with known physiological effects of opioid exposure and with no differences based on contingency. To control for litter effects, only 1-2 offspring per sex per litter were included in either the adult oxycodone or cocaine IVSA groups. The remaining pups were used in separate, unrelated studies not reported here. All behavioral data were collected across 5 cohorts; cohort was included as a variable in initial analyses and, as no significant cohort effects were detected, data were collapsed across cohorts for all reported analyses.

### Adult offspring IVSA

Adult male and female offspring were trained to self-administer either oxycodone (0.1 mg/kg) or cocaine (0.5 mg/kg) during short access (2 h) sessions on FR1 for 7 sessions, followed by FR5 for 5 sessions. All sessions were conducted in MedAssociates chambers as previously described, and sessions were conducted 5 days/week. Following FR5, animals were tested on PR for 3 sessions. For PR, the response requirement for each subsequent drug delivery was increased until the subject failed to meet a requirement within 30 min. The response requirement for the ith reinforcement was given by R(i)=[5e0.2i-5], with the first 10 requirements as follows: 1, 2, 4, 7, 9, 12, 15, 20, 25, 32. Active lever presses included all presses, including those during the timeout. The day after the final PR session, animals were returned to the chamber, but the drug syringe was replaced with a syringe containing saline as a measure of drug seeking.

### Statistical analyses and inclusion criteria

Most data were analyzed using analysis of variance (ANOVA). For the acquisition of IVSA using FR1 in females prior to mating and adult offspring, three-way repeated measures ANOVA with lever (active vs inactive) and session as within-subject factors and SA group as the between-subject factor was used. Maintenance of IVSA was analyzed using two-way repeated measures ANOVA with session as the within-subject factor and SA group as the between-subject factor. In adult offspring, acquisition and maintenance were analyzed separately in male and female offspring. Sex differences in drug seeking under extinction conditions were analyzed using two-way ANOVA with sex and SA group as between-subject factors. Due to significant positive skewness (Shapiro-Wilk *p* < 0.001), sex differences in motivated responding during PR were analyzed using a Generalized Linear Model (Gamma distribution, log link). Post hoc comparisons were conducted using appropriate corrections for multiple comparisons depending on the analysis. Significance was set at *p* < 0.05.

## Results

### Preconception and gestational self-administration

During oxycodone acquisition, three-way mixed ANOVA revealed significant main effects of lever (*F*_[__1,26__]_ = 21.94, *p* < 0.001) and SA group (*F*_[__2,2__6]_ = 11.77, *p* < 0.001), with a significant day x SA group interaction (*F*_[7.9__,__102.64]_ = 2.75, *p* = 0.009), lever x SA group interaction (*F*_[__2,2__6]_ = 16.00, *p* < 0.001), and day x lever x SA group interaction (*F*_[7.35__,__95.59]_ = 4.23, *p* < 0.001). The Oxycodone-Lead group demonstrated significant active versus inactive lever pressing beginning on session 4 (Fig. 1B), while neither Saline-Yoked nor Oxycodone-Yoked showed this difference. Beginning in session 4, Oxycodone-Lead active lever pressing was significantly higher than other SA groups.

For SA during gestation (Fig. 1C), there were significant main effects of lever (*F*_[__1,__26]_ = 31.60, *p* < 0.001) and SA group (*F*_[__2,__26]_ = 26.39, *p* < 0.001), and a significant lever x SA group interaction (*F*_[__2,2__6]_ = 35.04, *p* < 0.001). Across all days of pregnancy, the Oxycodone-Lead group had significantly higher active lever pressing compared to both Saline- and Oxycodone-Yoked females. Because animals were yoked, there were no differences in the number of infusions received preconception (data not shown) or gestation (Fig. 1D).

### Bodyweight gain and postnatal outcomes

Both Oxycodone-Lead and Oxycodone-Yoked females had reduced body weight gain compared to Saline-Yoked females beginning at the end of preconception acquisition and continuing throughout gestation. During acquisition (Fig. 1E), there was a main effect of day (*F*_[3.9,99.77]_ = 98.16, *p* < 0.001) and a day x SA group interaction (F_[28,364]_ = 4.75, *p* < 0.001), with both oxycodone groups weighing significantly less than Saline-Yoked at mating. Because oxycodone groups started pregnancy at lower weights, we calculated daily bodyweight changes across gestation to assess relative weight gain. Daily change (Fig. 1F), revealed main effects of day (*F*_[9.5,247.1]_ = 20.60, *p* < 0.001) and SA Group (*F*_*[2,26]*_ = 25.97, *p* = <0.001), and a day x SA Group interaction (*F*_[38,494]_ = 1.54, *p* = 0.02) with differences primarily on day 14 and the last 3 days of gestation. Total bodyweight gained during gestation showed a significant effect of SA Group (*F*_[2,26]_ = 26.16, *p* = <0.001; Fig. 1G) with both oxycodone groups gaining less weight than Saline-Yoked.

On PND1, all litters were weighed, culled to 8-10 pups with equal sex distribution and transferred to foster dams. Oxycodone infusions during gestation resulted in significantly smaller litters when compared to Saline-Yoked controls (SA Group, (*F*_[2,52]_ = 3.5, *p* = 0.037; Fig. 1H), reaching significance in the Oxycodone-Lead group (*p* = 0.04) and trending but not significant in the Oxycodone-Yoked group (*p* = 0.05). Individual pup weights showed clear differences between groups (SA Group, (*F*_[2,52]_ = 6.95, *p* = 0.002). Females weighed less than males (Sex ([F_[1,52]_ = 6.78, *p* = 0.01), but this effect was not further moderated by SA Group (sex by SA Group (F_[2,52]_ = 0.24, *p* = 0.78). Oxycodone-Yoked pups were significantly smaller than Saline-Yoked pups (*p* = 0.001), and there was a trend toward them being smaller than Oxycodone Lead pups (*p* = 0.06; Fig. 1I), while Saline-Yoked and Oxycodone-Lead pups did not differ (*p* = 0.33). We examined whether total bodyweight gain across pregnancy correlated with the dam’s total oxycodone intake. There was a significant negative correlation in the Oxycodone-Lead group (*r* = –0.63; *p* = 0.02) but not the Oxycodone-Yoked group (Fig. 1J). There was no correlation with the dam’s total oxycodone intake and offspring bodyweight on PND1 in either group (Fig. 1K).

### Adult offspring oxycodone self-administration

**Fig. 2:**
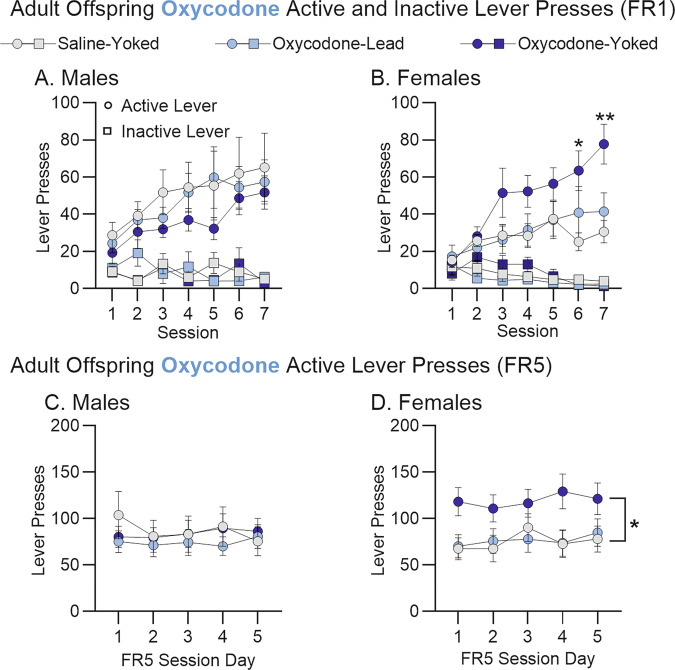
Adult offspring oxycodone self-administration. Active and inactive lever presses during FR1 acquisition in **A** male and **B** female offspring. Female offspring of Oxycodone-Yoked dams showed significantly elevated active lever pressing relative to both Saline-Yoked and Oxycodone-Lead female offspring by session 6. Active lever presses during FR5 maintenance in **C** male and **D** female offspring. Elevated active lever pressing persisted in Oxy-Yoked female offspring across all FR5 sessions. Data are expressed as mean ± SEM. *p < 0.05, **p < 0.01 vs. Saline-Yoked offspring.

All adult male offspring acquired the task as demonstrated by significant main effects on lever (*F*_[1,33]_ = 36.86, *p* < 0.001) and session (*F*_[2.7,90.3]_ = 3.78, *p* = 0.02) as well as a session x lever interaction (*F*_[2.5,85.7]_ = 6.86, *p* < 0.001; Fig. 2A), with no effects related to their dam’s SA group. All adult female offspring also acquired the task as indicated by significant effects on lever (*F*_[1,32]_ = 71.73, *p* < 0.001), session (*F*_[4.1,129.9]_ = 4.39, *p* = 0.002) and a session x lever interaction (*F*_[3.9,123.3]_ = 16.22, *p* < 0.001); however, females showed a significant effect of their dam’s SA group (*F*_[1,32]_ = 3.82, *p* = 0.03; Fig. 2B). This effect emerged over sessions (session x SA Group *F*_[8.1,129.9]_ = 2.01, *p* = 0.049) driven by increased active lever pressing specifically in female offspring of Oxycodone-Yoked dams (session x lever x SA Group *F*_[7.7,123.3]_ = 2.53, *p* = 0.015). This effect reached significance by session 6 (*p* < 0.05 as compared to Saline-Yoked) and by the final FR1 session, female offspring of Oxycodone-Yoked dams had higher active lever pressing compared to Saline-Yoked offspring (*p* < 0.001), and there was a trend toward significance when compared to Oxycodone-Lead female offspring (*p* = 0.055). Adult offspring were then moved to an FR5 schedule. No effects of the SA group were observed in males (Fig. 2C), while female offspring of Oxycodone-Yoked dams continued to demonstrate elevated active lever pressing (SA Group *F*^[2,32]^ = 3.6, *p* = 0.036; Fig. 2D), stable across the sessions.

### Adult offspring cocaine self-administration

**Fig. 3:**
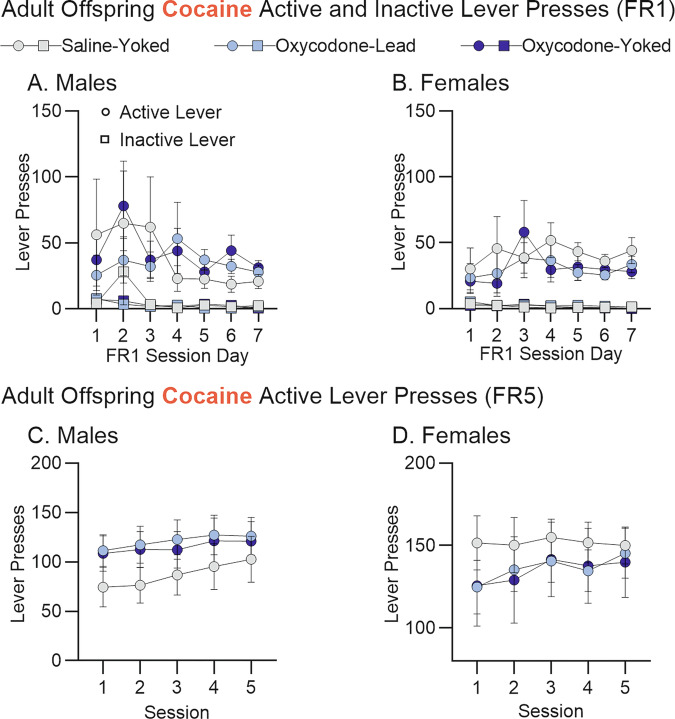
Adult offspring cocaine self-administration. Active and inactive lever presses during FR1 acquisition in **A** male and **B** female offspring. All groups acquired the task with no significant effects of dam SA group. Active lever presses during FR5 maintenance in **C** male and **D** female offspring. Males showed a significant increase in active lever pressing across sessions collapsed across groups; no SA group effects were observed in either sex. Data are expressed as mean ± SEM.

To determine whether the observed effects on Oxycodone IVSA in Oxycodone-Yoked female offspring generalized to other classes of drugs of abuse, we examined cocaine IVSA in adult male and female offspring. Male offspring acquired the operant task when responding on an FR1 schedule for cocaine with a main effect on lever (*F*_[1,31]_ = 25.19, *p* < 0.001; Fig. 3A), with no effects related to their dam’s SA group. Female offspring demonstrated the same pattern with a main effect of lever (*F*_[1,29]_ = 22.58, *p* < 0.001; Fig. 3B) but no other significant effects. Following transition to FR5, there was a significant effect of session (*F*[_2.6,81]_ = 3.92, *p* = 0.015) but no effect of SA Group or session x SA group interaction in males. Lever presses increased in sessions 4 and 5 compared to session 1 (both p’s < 0.05) collapsed across the SA group (Fig. 3C). Females showed stable active lever pressing across FR5 sessions (Fig. 3D) with no significant effects. Thus, unlike the IVSA pattern observed in response to oxycodone, no differences in responding for cocaine using fixed schedules were observed in either males or females.

### Sex differences in motivated responding for oxycodone and cocaine

**Fig. 4:**
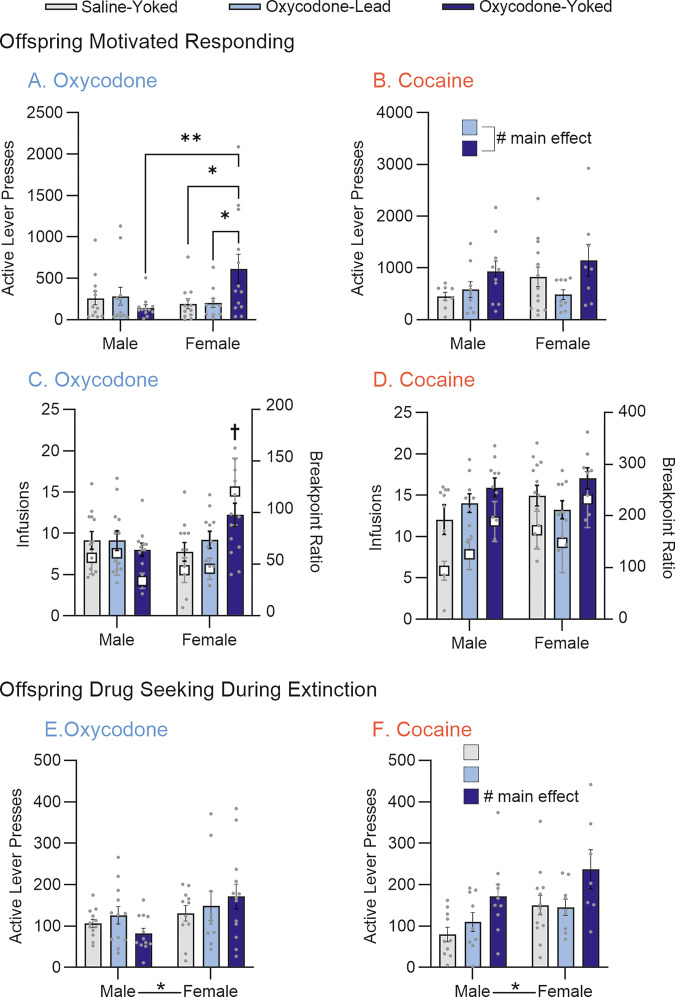
Sex differences in motivated responding and drug seeking. Average active lever presses across the three PR sessions for **A** oxycodone and **B** cocaine IVSA. Oxycodone-Yoked female offspring showed significantly higher motivated responding for oxycodone compared to all other groups. For cocaine, Oxycodone-Yoked offspring of both sexes showed significantly higher motivated responding compared to Oxycodone-Lead offspring. Average infusions earned (left y-axis, colored bars) and breakpoint ratio (right y-axis, open squares) across the three PR sessions for **C** oxycodone and **D** cocaine IVSA. Active lever presses on the first day of extinction following **E** oxycodone and **F** cocaine IVSA. Females showed higher drug seeking than males across groups following oxycodone IVSA. Following cocaine IVSA, Oxycodone-Yoked offspring of both sexes showed significantly elevated drug seeking compared to Saline-Yoked and Oxycodone-Lead offspring. Data are expressed as mean ± SEM. *p < 0.05, **p < 0.01. # significant main effect of SA group. † Female Oxycodone-Yoked breakpoint ratio significantly higher than Female Saline-Yoked, Female Oxycodone-Lead, and Male Oxycodone-Yoked (*p* < 0.05).

Next, we examined sex differences in the motivated responding for oxycodone and cocaine across the 3 PR sessions. Due to significant positive skewness in both datasets, data were analyzed using Generalized Linear Models with a Gamma distribution and log link function. For oxycodone PR active lever pressing, there was a significant sex x SA group interaction (Wald χ²_[2]_ = 13.21, *p* = 0.001) with no main effects (Fig. 4A). Post hoc analyses reveal significantly higher motivated responding in female offspring of Oxycodone-Yoked dams as compared to both Saline-Yoked (*p* = 0.02) and Oxycodone-Lead females (*p* = 0.02), as well as to Oxycodone-Yoked males (*p* < 0.01). No other sex differences were observed, indicating a sex-specific effect on motivated responding for oxycodone in Oxycodone-Yoked offspring. Consistent with this effect on lever pressing, a significant sex x SA group interaction was also observed for breakpoint ratio (Wald χ²_[2]_ = 13.03, *p* = 0.001), with Female Oxycodone-Yoked offspring achieving a higher breakpoint than Female Oxycodone-Lead (*p* = 0.048), Female Saline-Yoked (*p* = 0.039), and Male Oxycodone-Yoked (*p* = 0.012) groups (Fig. 4C). No significant effects were observed for infusions earned (all *p* > 0.05).

A different pattern was observed for motivated responding to cocaine. For active lever pressing, there was a significant main effect of SA group (Wald χ²_[2]_ = 8.47, *p* = 0.014) but no main effect of sex and no sex x SA group interaction (Fig. 4B). This effect was due to significantly increased active lever pressing in Oxycodone-Yoked offspring compared to Oxycodone-Lead offspring (*p* = 0.04) but not Saline-Yoked offspring (*p* = 0.11). No differences were observed between Oxycodone-Lead and Saline-Yoked offspring (*p* = 1.0). Thus, unlike the effects observed during oxycodone IVSA, Oxycodone-Yoked offspring, regardless of sex, are more motivated to obtain cocaine. No significant effects of sex, treatment, or their interaction were observed for infusions earned or breakpoint ratio during the cocaine PR session (all *p* > 0.05; Fig. 4D), although breakpoint tended to be higher in Oxycodone-Yoked as compared to Oxycodone-Lead offspring (*p* = 0.08), consistent with the lever pressing findings.

### Sex differences in drug seeking under extinction conditions following either oxycodone or cocaine

For initial drug seeking under extinction conditions following oxycodone IVSA, there was a significant main effect of sex (*F*_[2,64]_ = 6.09, *p* = 0.02) but no effect of SA group and no sex x SA group interaction. Females had higher levels of drug seeking on the first day of extinction, compared to males collapsed across all groups (Fig. 4C). Drug seeking following cocaine IVSA revealed a significant main effect of SA group (*F*_[2,53]_ = 6.3, *p* < 0.01) and sex (*F*_[1,52]_ = 7.01, *p* = 0.01) with no significant interaction. The SA group effect was due to significantly higher drug seeking in Oxycodone-Yoked offspring compared to both Oxycodone-Lead (*p* = 0.02) and Saline-Yoked offspring (*p* < 0.01; Fig. 4D). Thus, while females demonstrated the expected increase in drug seeking when compared to males, offspring of Oxycodone-Yoked dams had elevated drug seeking regardless of sex. These data indicate that non-contingent exposure to oxycodone during fetal development enhances motivated responding for oxycodone specifically in adult female offspring and drug seeking for cocaine in both sexes.

### Correlations between dam intake and offspring IVSA

**Fig. 5:**
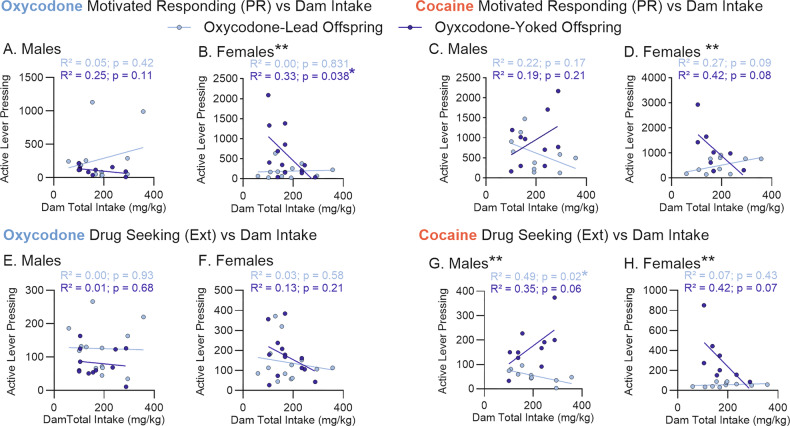
Correlations between dam gestational oxycodone intake and adult offspring IVSA. Pearson’s correlations between the cumulative dam oxycodone intake during pregnancy and average PR active lever pressing in **A** oxycodone male and **B** oxycodone female offspring, and cocaine PR active lever pressing in **C** male and **D** female offspring. Correlations between dam intake and drug seeking during extinction for **E** oxycodone males, **F** oxycodone females, **G** cocaine males, and **H** cocaine females. A significant negative correlation between dam intake and motivated responding for oxycodone was observed in Oxycodone-Yoked female offspring. Higher dam intake positively correlated with cocaine drug seeking in Oxycodone-Yoked male offspring. No significant correlations were observed in Oxy-Lead offspring of either sex. *p < 0.05, **p < 0.01.

One strength of the oxycodone self-administration model is the range of drug intake, which models divergent levels of use. We conducted sex-specific Pearson’s correlations between dam cumulative oxycodone intake during pregnancy and average PR and drug seeking during extinction for both oxycodone and cocaine in Oxycodone-Lead and Oxycodone-Yoked animals (Fig. 5). Higher dam intake negatively correlated with motivated responding for oxycodone in Oxycodone-Yoked female offspring (R^2^ = 0.33; *p* = 0.038; Fig. 5B). When examining cocaine drug seeking during extinction, higher dam intake positively correlated with responding in Oxycodone-Yoked males (R^2^ = 0.49; *p* = 0.02; Fig. 5G), with no significant correlation observed in Oxycodone-Yoked females (R^2^ = 0.42; *p* = 0.07; Fig. 5H). No significant correlations were observed in Oxycodone-Lead offspring of either sex. These findings suggest that the level of non-contingent, but not contingent, prenatal oxycodone exposure shapes drug motivation in adult offspring in a sex- and drug-specific manner.

## Discussion

### Maternal control over drug intake shapes offspring vulnerability

This study revealed a clear dissociation between contingent versus non-contingent oxycodone exposure during pregnancy on offspring substance use vulnerability. Despite similar total oxycodone exposure, only offspring from dams receiving non-contingent administration showed enhanced intake under FR1, motivated responding, and drug-seeking behaviors. Notably, the effects of non-contingent exposure were sex-dependent, with female offspring of Oxycodone-Yoked dams exhibiting enhanced intake and motivated responding specifically for oxycodone, while offspring of both sexes displayed elevated cocaine seeking under extinction conditions. This suggests that maternal control over drug intake, rather than opioid exposure alone, is critical in determining long-term outcomes. While previous studies have demonstrated that prenatal opioid exposure can impact reward-related behaviors [21, 22]. Our findings indicate that a lack of maternal control may be a key factor driving these effects.

The selective effects in Oxycodone-Yoked offspring, despite equivalent drug exposure, suggest that direct pharmacological actions of oxycodone on fetal development may not be the primary driver of enhanced substance use vulnerability. Instead, the loss of control over drug administration may induce distinct physiological responses in Oxycodone-Yoked dams that impact fetal development. This is supported by our observation of reduced birth weight, specifically in Oxycodone-Yoked offspring. Non-contingent delivery of opioids could lead to different stress responses, including altered hypothalamic-pituitary-adrenal axis function [23], or disrupted circadian patterns [24, 25], compared to dams with controlled intake, thereby influencing the development of reward circuits in the fetus [26, 27].

### Sex-specific effects and differences based on drug class

Although sex differences in addiction vulnerability are well-established in preclinical models [28], prenatal opioid exposure studies have historically focused predominantly on male offspring [4, 6]. While a growing body of work over the past decade has incorporated sex as a biological variable [5], many female-specific effects in response to prenatal substance exposure remain largely uncharacterized. The sex-specificity of the current effects, particularly with regard to oxycodone versus cocaine vulnerability, suggests divergent neurodevelopmental consequences of non-contingent prenatal exposure. Female offspring of Oxycodone-Yoked dams showed enhanced motivation specifically for oxycodone, while both sexes exhibited elevated cocaine seeking. Notably, the relationship between maternal intake and offspring behavior differed by both sex and drug. In females, lower levels of maternal oxycodone exposure were associated with higher motivated responding for both oxycodone and cocaine. That said, as only one dose of oxycodone was evaluated in offspring, we cannot determine whether the enhanced responding observed in Oxycodone-Yoked females reflects an increase in overall motivation for oxycodone or a change in oxycodone’s potency; future dose-response studies will be necessary to distinguish between these possibilities.

The negative correlation between maternal oxycodone intake and offspring motivated responding in Oxycodone-Yoked females was unexpected and suggests a complex dose-dependent relationship. One possible explanation centers on maternal stress. Because yoked dams lack control over drug delivery, opioid administration itself may function as a stressor [23]. At lower doses, non-contingent exposure may be insufficient to suppress the HPA axis response, resulting in sustained elevation of maternal stress hormones throughout pregnancy. At higher doses, opioid-mediated suppression of the HPA axis [29] may paradoxically buffer the dam, and by extension the fetus, against the effects of repeated uncontrollable exposure. This dose-dependent modulation of maternal stress and its downstream effects on fetal glucocorticoid exposure could underlie the inverse relationship observed in female offspring.

### Limitations and translational implications

Previous studies examining prenatal opioid exposure have typically used experimenter-administered opioids [4–6]. This may explain why these studies often report increased substance use vulnerability, as a lack of maternal control over drug administration could be a factor. Our finding that offspring of dams with controlled oxycodone intake showed no enhancement of drug-taking or seeking behaviors suggests that previous findings of increased risk may be related in part to patterns of use or interactions with stressors rather than opioid exposure per se. Indeed, these data align with those of Smith et al [30] using a similar prenatal oxycodone IVSA model, who reported no significant differences in oxycodone conditioned place preference in male or female offspring of oxycodone self-administering dams. That said, several limitations of the current study should be acknowledged. All offspring were cross-fostered at birth to drug-naïve dams. This was done to minimize potential confounds of altered maternal care, as we previously demonstrated significantly delayed maternal retrieval in oxycodone self-administering dams using a similar model [20]. Thus, disrupted maternal care in opioid-exposed dams remains another potential modifier of offspring risk that warrants future study. Furthermore, prenatal exposure in rats occurs during a developmental period equivalent to the first two trimesters in humans [31]. It is unknown whether further exposure during subsequent neurodevelopment would result in additional effects. Therefore, while maternal control represents one critical factor influencing offspring outcomes, other variables, including exposure that continues during postnatal neurodevelopment and the impact of altered maternal care, may also play an important role in long-term outcomes.

The current findings have potential translational relevance for understanding how patterns of opioid use during pregnancy may influence offspring outcomes. The central manipulation in the current study raises the question of whether the degree of maternal control over opioid intake during pregnancy plays a meaningful role in mediating some of the long-term effects of fetal opioid exposure. Notably, the absence of enhanced substance use vulnerability in offspring of Oxycodone-Lead dams suggests that the pattern and context of opioid use during pregnancy, not simply exposure per se, may be an important determinant of offspring risk. This has potential relevance for ongoing discussions about the management of opioid use during pregnancy, including the use of medications such as methadone and buprenorphine [32, 33], though we acknowledge that the motivational, neurobiological, and social context of opioid use in pregnant women involves many factors that cannot be fully captured in a preclinical model. The sex-specific effects observed here further underscore the importance of including sex as a biological variable in preclinical research.

In conclusion, this study demonstrates that prenatal oxycodone exposure impacts offspring substance use vulnerability, dependent on both maternal control over intake and offspring sex. The critical role of maternal agency suggests that previous findings of enhanced vulnerability may be specifically related to patterns of use rather than opioid exposure alone. The complex, sex-specific relationships between maternal intake and offspring behavior highlight the importance of considering both sex and dosing patterns when evaluating developmental risk. These findings provide new insights into factors influencing substance use vulnerability following prenatal opioid exposure and suggest new directions for preclinical research and potentially for translation into novel intervention strategies for women.

## Data Availability

The data that support the findings of this study are available from the corresponding author upon request.
